# Exploring the Use of Technology for Sexual Health Risk-Reduction among Ecuadorean Adolescents

**DOI:** 10.5334/aogh.35

**Published:** 2019-04-15

**Authors:** Christopher Reynolds, Melissa A. Sutherland, Iván Palacios

**Affiliations:** 1 Decker School of Nursing, Binghamton, NY, US; 2 Colegio de Ciencias de la Salud, Universidad San Francisco de Quito, Quito, EC

## Abstract

**Background:**

There is a lack of sexual health knowledge and resource access among youth in Latin America, along with rising rates of teenage pregnancy and STD transmission.

**Objectives:**

To determine baseline sexual health knowledge and the acceptance of a technology based sexual health risk-reduction program among Ecuadorean adolescents.

**Methods:**

We used mixed methods to determine the sexual health knowledge and practices, and technology use among 204 adolescents from two schools in Cumbayá and Lumbisí, Ecuador. Quantitative data was collected through surveys and qualitative through single-gender focus groups.

**Findings:**

Nearly every participant (96.6%) expressed interest in a sexual health education program using technology and social media. A majority of participants indicated that they consulted parents (58.3%) regarding sexual health questions. Only a few participants had access to physicians outside of appointments (3.9%), and most desired more sexual health information (87.3%). Although approximately one-quarter of participants were sexually active (27%), most lacked baseline knowledge regarding contraceptives and STDs. Facebook (91%) and WhatsApp (53%) were the most frequently used and requested social media for an educational program. Students indicated a strong desire to be involved in the design stages of a sexual health risk-reduction program, rather than use a pre-established program.

**Conclusions:**

There is strong interest in a technology based sexual health risk-reduction program through Facebook and WhatsApp, which could establish communication between health providers and Ecuadorian youth to disseminate health information and answer private inquiries. Findings from this study, the first of its kind among South American adolescents, introduces a novel idea: involving participants from initial design stages of a text-messaging health education program. Future studies should focus on engaging families as well as physicians’ willingness to participate.

**Implications and Contributions:**

This paper is the first acceptability study of a technology based sexual health risk-reduction program among low-income South American adolescents. Findings enhance understanding of pregnancy and STD prevention interventions by demonstrating participants’ desire for self-design and implementation, and highlight their importance through a lack of baseline adolescent sexual health knowledge.

Lack of sexual health education is a barrier to health access, empowerment and autonomous health decisions among adolescents, as evidenced by the number of pregnancies and sexually transmitted diseases (STDs) annually diagnosed. Worldwide, 16 million girls aged 15–19 become pregnant each year [1,2,3], with 95% occurring in low and middle-income countries [1,3,4,5]. Adolescent pregnancy can present danger to the mother and child, including depression [6], unsafe abortion, preterm delivery, and child stunting [1,7]. Poor adolescent mothers are especially at-risk for early exit from educational programs [1,8,9], social stigmatization [10,11,12], partner violence [13,14], and cyclic poverty [1,9].

Latin America and the Caribbean is the region with the highest adolescent pregnancy rate after Sub-Saharan Africa, and the only in which adolescent fertility has increased in recent decades [14]. Projections indicate that by 2020, adolescent fertility in Latin America will be highest in the world and remain stable until 2100 [14]. Ecuador yields the highest rate of adolescent pregnancy in South America at just over 20% [16]. Among poor communities, this figure reaches levels of 30% [17], and half of Ecuadorean women aged 18–24 have at least one child [16]. Though Ecuador has made significant advances by halving the adolescent pregnancy rate over the last forty years, these rates have slightly increased during the last decade [17].

Lack of sexual health programs is also linked to STD transmission [18,19]. Ecuadorean youth are especially at-risk, as more than half of newly diagnosed STDs in Latin America are among adolescents [2], and societal stigma can delay detection and treatment [20,21]. Despite increased contraceptive access and use in Ecuador over the last two decades [16], rates of adolescent pregnancy and STD transmission have not significantly declined, and in some years have actually risen [15,22].

Even with increased access to health resources, many youths have difficulty accessing and communicating with their healthcare providers [23]. With the stigmatization of sexual health conversations, taboo social perceptions [19,21], and a lack of mainstream programs focused on health empowerment [18], many Ecuadorian adolescents lack essential sexual health knowledge and accurate information sources [15].

Technology could be useful in disseminating sexual health information to Ecuadorian youth. Latin America has a rapidly growing internet population, with adolescents using social media at the highest rates [24]. Previous research has shown youth prefer trusted technology rather than face-to-face meetings, due to its anonymity and privacy [23,25], and multiple programs in the United States and Africa have shown the efficacy of technology implemented health education [4,25,26,27,28,29,30]. Despite these successes, no studies have examined a low-income South American community regarding the acceptability of an adolescent sexual health risk-reduction program, nor engaged participants before program implementation.

Before designing a program, it was important to determine student interest, technology use and gaps in sexual health knowledge. We believed it useful to engage adolescents beginning with the design stage to empower them as agents in their own care. We investigated sexual health knowledge and technology use among adolescents in Cumbayá and Lumbisí, Ecuador; two regions outside of Quito. This setting was ideal because of the disproportionately high adolescent pregnancy rates. Specifically, this study sought to: (a) examine access to sexual health information; (b) determine baseline sexual health knowledge; and (c) identify the acceptability of a technology based sexual health risk-reduction program among adolescents.

## Methods

This mixed methods study was completed between September and November of 2016 as part of the Latitude-0 Ecuador Research Initiative of the Universidad San Francisco de Quito (USFQ), in collaboration with USFQ Medical School. Two low-income schools in Cumbayá and Lumbisí, Ecuador were selected as data collection sites. All participants spoke Spanish, with some being bilingual in Kichwa. Study procedures were approved by the Comité de Ética de Investigación en Seres Humanos at USFQ (IRB), the participating schools, and supervisory school districts prior to data collection.

### Quantitative

Sexual health knowledge, attitudes and practices, information sources, and technology use were collected via a self-administered, pen and paper survey. The survey consisted of three sections and 26 questions. Section one covered socio-demographic characteristics (e.g., gender, age, race). Section two included questions about technology access (e.g. cell phone), social media forms, and usage frequency. Access to cellular phones was assessed with yes or no questions, and social media frequency was determined by ranking. Section three focused on sexual health knowledge, attitudes and practices.

The preliminary questionnaire was reviewed by native Spanish speakers, and pre-tested by 20 youth to identify problems with phrasing and flow.

Letters to parents were distributed in three rounds with the purpose to introduce families to the project and obtain parental consent for participation. Since parental approval was necessary, selection bias of participants from families more willing to engage sexual health topics was possible. Consent was obtained from 252 students. In the first round of consent collection, 48 forms contained illegible signatures, and these students were not identified. Forms were altered to include student’s printed name and grade level for accurate identification in the final two rounds of consent collection. The response rate at school 1 was 17.4% and school 2 was 5.3%.

School administrators assisted with data collection by calling students with parental consent from class to complete the 15-minute survey. Three rounds of quantitative data collection took place resulting in a total of 204 participants. Medical students served as research assistants and answered participants’ questions.

Survey data were entered into Excel, examined and cleaned. Statistical analyses were conducted using SPSS v.20 and statistical significance was set at a value of p ≤ 0.05. Data and distributions were examined for outliers, abnormal values, missing data, and normality. Analyses by site and gender were conducted. Mean and standard deviations for continuous variables, and percentages for nominal data were determined.

### Qualitative

Following quantitative data analysis, three focus groups were conducted with participants from school 1. Two female groups of 11–12 (group 1) and 13–14 years of age (group 2), and one male group of 13–14 years of age (group 3) constituted each group. Criteria for selection were groups between 6 and 12 adolescents of the same grade level and gender. Participants were randomly selected by school administrators from students with parental consent. A semi-structure script was developed to extrapolate information from the questionnaire, and was preapproved by the IRB at USFQ. Question topics included: technology use, after-school activities, perception of opposite gender’s sexual health interest, and sources for health knowledge. Focus groups allowed participants to suggest ideas for design of a sexual health risk-reduction program. Probing questions were used to encourage elaboration from participants. The focus groups were moderated by researchers, audio recorded and notes were taken in Microsoft Word.

During qualitative data analysis, audio tapes were transcribed verbatim into Word. An audit trail was established that consisted of tapes, notes, and transcripts. Transcripts were read multiple times to achieve immersion and identify recurrent themes. The method of qualitative data analysis was content analysis [31].

## Results

### Quantitative

#### Sample Characteristics

The sample was 46.1% female and 52.5% male (Table 1). The age ranges (11–18 years) and mean age (14.9 years) was the same for male and female participants. Age of participants between schools was statistically significant (p-value = 0.003); the mean age of participants at school 1 was 14.7 (SD 1.96) and 15.8 years at 2. (SD 1.16).

**Table 1:** Demographic Characteristics and Sexual Health Knowledge of Schools 1 and 2.

	Total	School 1	School 2

**Number of participants; *n* (%)**	204	174 (85.3%)	30 (14.7%)
**Age; years (SD)**	14.8 (±1.8)	14.7 (±2.0)	15.8 (±1.2)
Gender; *n* (%)**			
Female	94 (46.1%)	92 (52.9%)	2 (6.7%)
Male	107 (52.5%)	79 (45.4%)	28 (93.3%)
**Race; *n* (%)**			
Mestizo	192 (94.1%)	166 (95.4%)	26 (86.7%)
Indigenous	4 (2.0%)	4 (2.3%)	0 (0.0%)
White	1 (0.5%)	1 (0.6%)	0 (0.0%)
Afroecuadorian	1 (0.5%)	1 (0.6%)	0 (0.0%)
Other	2 (1.0%)	0 (0.0%)	2 (6.6%)
**Phone access; *n* (%)**			
Has personal phone	134 (65.7%)	63 (67.0%)	71 (66.4%)
Has smartphone	80 (39.2%)	33 (35.1%)	47 (43.9%)
Can receive text messages	139 (68.1%)	116 (66.7%)	23 (76.7%)
**Sexual Activity/Knowledge; *n* (%)**			
Is sexually active	54 (26.5%)	35 (20.1%)	19 (63.3%)*
Is sexually active, >15 years old	2 (1.0%)	2 (1.1%)	0 (0.0%)
Is sexually active, <15 years old	52 (44.4%)	33 (19.0%)	19 (65.5%)*
Access to contraceptives	77 (37.7%)	57 (32.8%)	20 (66.6%)**
Always uses protection during sex	35 (39.8%)	25 (39.1%)	10 (41.7%)
Sometimes uses protection	27 (30.7%)	17 (26.6%)	10 (41.7%)
Never uses protection	26 (29.5%)	22 (34.4%)	4 (16.7%)
Knows it is possible to prevent pregnancy	172 (84.3%)	144 (82.8%)	28 (93.3%)
Knows how to prevent pregnancy	154 (75.5%)	134 (77.0%)	20 (66.6%)
Could list at least one way to prevent pregnancy	170 (83.3%)	147 (84.5%)	23 (79.3%)
Could list multiple ways to prevent pregnancy	95 (46.6%)	84 (48.2%)	11 (37.9%)
Access to STD information (HIV, Zika, etc.)	131 (64.2%)	111 (63.8%)	20 (66.6%)
Correctly identified how STDs are transmitted	73 (35.8%)	66 (37.9%)	7 (23.3%)

*n* = number, % = percent, SD = standard deviation, STDs = sexually transmitted diseases. * p < .05. ** p < .01. Cumbayá and Lumbisí, Ecuador, November 2016.

Gender between schools was significant (p < 0.001), as 93.3% of the school 2 sample was male. After age 13, school 2 is all male. Approximately 94% of participants self-identified as mixed race (mestizo), 2% as indigenous, and the remaining 4% as white, Afro-Ecuadorean, or other. Differences were noted in the socioeconomic status of the students from school 1 and 2. School 1 is a public school with 1,000 students from low-income families. School 2 is 95% male and is a private secondary school of 550 students from families with more resources.

Nearly 66% of the participants (n = 134) had a personal cell phone, though only 39.2% owned a smartphone. Slightly more respondents could receive text messages (68.1%, n = 139). Across schools, participants reported similar rates of phone ownership.

Nearly 27% (n = 54) reported being sexually active, with the mean age of first sex at 14.9 (SD 1.4). There was a large range (9 to 17 years old) in self-reported age of first sexual experience. Among participants younger than 15 years old, only 1% reported being sexually active, while 44% of those 15 to 18 years old reported being sexually active.

#### Sexual health knowledge, attitudes, and practices

Only 77 participants (37.7%) reported being able to access contraceptives, though adolescents at school 2 were significantly more likely to report access than those at school 1 (p = 0.009). Reported contraceptive use was low, with only about 40% of students reporting that they “always used protection during intercourse”, and nearly 30% of participants reported “never.” Most reported that they knew it was possible to prevent pregnancy. More than 80% identified one method of contraception, but less than half could name multiple. Although nearly 65% of the participants reported access to information about STDs, including HIV and Zika, only about 35% correctly identified how STDs are transmitted.

#### Sexual health resources

Nearly 85% (n = 173) of participants reported having received sexual health education (Table 2), with female participants more likely than male participants (p = 0.006). The vast majority (87.3%) desired more information, and females were significantly more likely to want more information than their male counterparts (p = 0.001).

**Table 2:** Desired Information and Sexual Health Access, male/female.

	Total	Male	Female

**Received sexual health education; *n* (%)**	173 (84.8%)	84 (78.5%)	89 (94.7%)**
**Want more sexual health information *n* (%)**	178 (87.3%)	82 (78.1%)	91 (96.8%)**
**Persons consulted regarding sexual health questions; *n* (%)**			
Parents	128 (62.7%)	59 (55.1%)	67 (71.3%)*
Friends	38 (18.6%)	22 (20.6%)	16 (17.0%)
Brother/sister	25 (12.3%)	16 (15.0%)	8 (8.0%)
Doctor	17 (8.3%)	9 (8.4%)	8 (8.5%)
Teacher	7 (3.4%)	4 (3.7%)	3 (3.2%)
Other (Family members, internet, no one)	21 (10.3%)	10 (9.3%)	11 (11.7%)
**Who did you consult the last time you had a question about sexual health? *n* (%)**			
Parents	119 (58.3%)	59 (55.1%)	60 (63.8%)
Friend	16 (7.8%)	11 (10.3%)	5 (5.3%)
Sibling	15 (7.4%)	10 (9.3%)	5 (5.3%)
No one	10 (4.9%)	8 (7.5%)	2 (2.1%)
Personal investigation (literature, internet, health talk)	10 (4.9%)	4 (3.7%)	6 (6.4%)
Doctor	8 (3.9%)	1 (0.9%)	7 (7.4%)
Teacher	5 (2.5%)	2 (1.9%)	3 (3.2%)
Aunt/Uncle	5 (2.5%)	4 (3.7%)	1 (1.1%)
Other (Family members)	16 (7.8%)	12 (11.2%)	4 (4.3%)
**Comfortable asking parents a sexual health question *n* (%)**			
Always	33 (16.2%)	14 (13.1%)	18 (19.1%)
Sometimes	104 (51.0%)	71 (66.4%)	62 (66.0%)
Never	37 (18.1%)	37 (18.1%)	14 (14.9%)
**Easy access to physician for sexual health question *n* (%)**			
Always	28 (13.7%)	13 (12.1%)	15 (16.0%)
Sometimes	106 (52.0%)	52 (48.6%)	52 (55.3%)
Never	69 (33.8%)	42 (39.3%)	26 (27.7%)
**Can speak to a physician outside of an appointment *n* (%)**			
Always	8 (3.9%)	4 (3.7%)	4 (4.3%)
Sometimes	63 (30.9%)	31 (29.0%)	32 (34.0%)
Never	126 (61.8%)	68 (63.6%)	58 (61.7%)
**Would you participate in a sexual health education program through technology, which connects you with a doctor to answer your questions and provide information? *n* (%)**			
Yes	130 (63.7%)	62 (58.5%)	65 (70.7%)
Occasionally	32 (15.7%)	22 (20.8%)	10 (14.1%)
Maybe	32 (15.7%)	19 (17.9%)	13 (14.1%)
Never	7 (3.4%)	3 (2.8%)	4 (4.3%)

*n* = number, % = percent. * p < .05. ** p < .01. Cumbayá and Lumbisi, Ecuador, November 2016.

Our sample reported that parents (62.7%), friends (18.6%) and siblings (12.3%) were sources of sexual health information. Female participants were more likely to consult their parents (p = 0.018), whereas males were more likely to seek advice from friends or siblings.

Less than one-fifth of students (16.2%) indicated they would “always be comfortable” to ask their parents a sexual health question, while a majority (51%) reported only feeling comfortable “sometimes”. Many participants reported “never feeling comfortable” asking a question (n = 37).

#### Access to health professionals

Adolescents reported a lack of access to health professionals to consult regarding their health questions. Less than 15% (n = 28) of participants reported “always having easy access to a doctor”. Only 4% (n = 8) reported to “always be able to speak with a doctor outside of planned visits,” and 126 respondents (61.8%) reported “never”. Only 8% responded “doctor” from a multi-selection (parents, doctor, friends, sibling, teacher) as someone they contact and trust regarding their sexual health questions. Only 8 participants mentioned a non-relative medical professional as the person they consulted the last time they had a sexual health related question. There were more adolescents who reported not asking anyone (n = 10), than those who consulted a physician.

#### Desire for program

When asked about a sexual health risk-reduction program through technology, almost 80% (n = 162) of participants said they would “definitely” or “occasionally” utilize such a program, and less than 4% (n = 7) indicated they would “never.” Response rates were similar across gender. Of the 134 participants with a personal cell phone, (Table 1), nearly 80% (n = 107) provided contact information for a follow-up study for pre-testing of the program. More than 30% of participants (n = 65) wrote a sexual health question in the write-in section. Nearly 80% (n = 160) wrote-in information they would like such a program to provide, and approximately 40% of participants (n = 82) provided suggestions for program design. Requested topics included pregnancy prevention, STDs, and self-care, while most suggestions for design focused on relatability, “keeping it light”, and supplementary health lectures by medical students (Table 3).

**Table 3:** Focus group topics, Responses and Relevance to Quantitative Data and Program Design.

Topic	Researcher Observations and Participant Responses (“”)	Relevance

Sexual health knowledge	Received a one-time health talk from the local clinic about pregnancy prevention. Little information from the school about STDs, contraceptives, and self-care. Females identified many methods of contraception, males only knew of condoms. “In natural sciences, they explained a little about these themes…including puberty, how to not become pregnant, and the diseases we could catch.”	Clarified quantitative data, that though >80% received sexual health education, it is not sufficient or continual. Revealed a gap with males in not only contraceptive access, but also knowledge.

Information sources	Only 4 of 32 have consulted the school psychologist about sexual health. There is no doctor on staff. “I have spoken a little [with my parents] but really we haven’t spoken that much. With my mom we talk about it all but not that much with my dad.” “Yes, we’ve spoken a little. Mostly things like how to take care of myself.” “I talked to my mom during my last menstruation. She told me how to take care of it and I have talked to her about that.”	Confirms that parents are the most common source of sexual health knowledge. Reveals that most ask their parents only about topics of puberty, not sexual practices.

Information desires	All participants reported they have not received sufficient sexual health information. 27 of 32 participants reported they would like to receive more sexual health information. Desired biweekly lectures from medical students to discuss sexual health. Participants recommend the program be “cool, fun and relaxed” and “relatable to students.” “They have not given us sufficient information.” “I would like to receive information about everything we are talking about. All of it! In my house, neither, we don’t discuss these topics. My parents tell me it’s important to know about these things, but we have never discussed them.” “I would like more information about how to take care of myself. How to prevent pregnancy.”	Participants desire more sexual health information; confirms quantitative data. Desire pregnancy prevention, STD and self-care information; confirms write-in responses. Program design suggestions confirm write-in responses, that students want the program to be relatable and fun.

Technology preference	Younger groups prefer Facebook for its accessibility. Older group prefers WhatsApp for its privacy. Preference for WhatsApp over text messaging. There is a wide range in cell phone access according to age. All participants had computer access. 31 of 32 participants have Facebook and use Messenger feature. Facebook Messenger was identified as appropriately private. “I think yes, I would like access to a Facebook page with doctors’ cell phone numbers because this would serve some people without phones. And they could decide what they want to see and others they don’t.” “I would use the cell phone messaging because it is much more private.” “WhatsApp is better because…I don’t know…I use it more.”	Preference for WhatsApp over text messaging for asking private questions. Suggests a joint-system with Facebook to upload public information and WhatsApp for private inquiries. Privacy concern differences, with older participants more conscious of anonymity. Difference in technology access and sexual behaviors suggest age-dependent curriculum. High Facebook usage confirms quantitative data.

Other gender’s interest	Female participants enthusiastically responded “no” when asked if their male counterparts have the same interest as them in sexual health information. “Boys are not interested in such topics [on sexual health].” “Boys only look for mischievous things.” Male participants refused to answer the question about females, stating “there is not confidence to say.”	Female participant perception agrees with quantitative responses that females are more interested in sexual health topics than male participants. Lack of knowledge regarding the other gender’s sexual health interest shows little communication between each population.

After school activities	Most participants were excited to spend afternoons at a sexual health lecture. Male participants used the computer an average of 2 hours per day, while a little more than half had daily exercise. Less than half of female participants reported daily exercise. “I usually go home, maybe watch television, talk on the phone or take a nap.”	Lack of scheduled activities and participant recommendations suggest hosting afternoon medical student lectures. Future studies could focus on designing recreational and exercise opportunities.

Fear to speak	Younger participants were much less willing to talk. Participants checked for social cues from other participants before responding. The male group was particularly hesitant. Responses required calling individual participants by name, which usually led to one-word responses. Later conversations with the school psychologist confirmed a lack of confidence among students, and a classroom dynamic to encourage passive learning.	Older adolescents could be abler in their health. Male participants could require a specifically designed program to engage them. Studies could focus on classroom empowerment. Empowerment and autonomy should be part of a well-designed intervention.

Cumbayá, Ecuador, November 2016.

**Figure 1:**
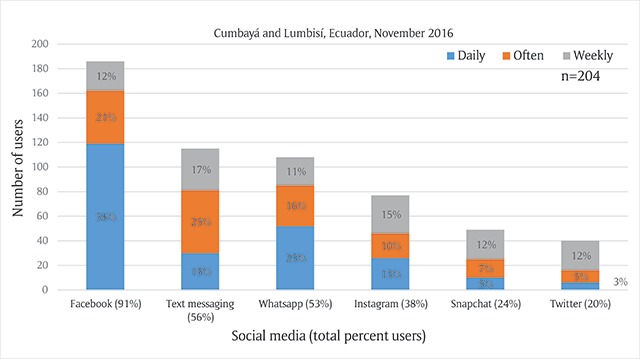
Total Participants and Frequency of Social Media Use.

Facebook was the technology with the greatest number of users and most frequent use, with around 58% (n = 119) of participants reporting daily use, and only 4.4% (n = 9) reporting never using Facebook (Figure 1). Second in user number, text messaging (n = 131) had 16 more than WhatsApp. WhatsApp was second most frequently used with 25.5% daily users compared to text messaging’s 14.7%, but also 43.6% of participants never used WhatsApp. Instagram, Snapchat, and Twitter were used significantly less than the three other modes of technology, with daily uses at 13%, 5%, and 3%.

### Qualitative

#### Sexual health

Focus group responses supported and clarified the quantitative survey data. Despite high numbers reporting having received sexual education (84.8%), participants had only received a one-time lecture from the local clinic. As one participant said:

“At the beginning of last year we had a lecture about how to protect ourselves, different preservatives, and pregnancy prevention from the health center…after that talk they have not returned for another one.”

There was no continual school or clinic-sponsored sexual health education program. Aside from this one-time talk, students receive no formal sexual health education.

#### Information sources

The majority of participants consulted parents regarding sexual health questions, but there were barriers to these discussions:

“I’ve spoken a bit with my mom about menstruation and how not to become pregnant. It’s something complicated to bring up. They say we should study, and that we shouldn’t involve ourselves in these types of things.”

Nor was there much conversation within the school. The school has a female psychologist on-staff, but few have used her as a resource (Table 3). There was no doctor on staff. When questioned about the other gender’s perception of sexual health, female participants reported low interest among their male counterparts. As one female stated:

“In some cases, I think [boys] would ask questions [about sexual health] because this information would help them with their girlfriends. But that is not all of them, there are only a few.”

Worth noting was that male participants refused to answer the question and were hesitant to answer most others, despite questioners’ enco-uragement.

Every student in the focus group felt that they had not received sufficient sexual health information:

“Yes I want more information because some topics nobody has told us about, and sometimes the people you ask don’t have the answers or you can’t speak to parents about it.”

When a technology based sexual health program was explained to the students, all indicated a desire to participate. Most said they wanted “all the information,” but pregnancy prevention and self-care were highlighted topics.

All participants had computer access, and most used Facebook. The majority of older participants had personal cell phones, while less than half in the younger groups did. Group 2 expressed privacy concerns, and stressed complete anonymity, though privacy concern was not raised by the younger groups.

Most students reported having no recreational activities after classes, and almost all requested supplementary sexual health talks by medical students. Participants recommended the program be “cool, fun and relaxed” and “relatable.” There was a clear social fear in answering most questions, especially with the male participants and even among non-stigmatized topics.

## Discussion

This study is the first in Latin America to examine adolescent attitudes to a technology based sexual health program. Findings revealed four main themes. First, there is a strong desire among Ecuadorean adolescents for a technology based sexual health risk-reduction education program. Quantitative results, the many qualitative write-ins, and participants who provided contact information for a pre-trial showed substantial interest for participation in program design. These findings alongside successful community-based-health-programs [32] suggest that participants should be engaged in the program’s planning.

Second, results showed a lack of knowledge and reliable sources of accurate sexual health information. There was a gap in STD knowledge and contraceptive usage and access. While most understood the basics of pregnancy prevention, many participants still requested related information. These topics should be integral to a proposed program. Participants lacked health information sources, as most consult their parents, yet feel uncomfortable discussing topics outside of puberty. Few participants had access to their physician outside of appointments, and neither school offered continual sexual health education. Along with participant desires, the lack of knowledge and information sources suggests the need for an education program as a reliable source for health information. The higher rate of contraceptive access at school 2 supports research showing the relationship between socioeconomic status and health resources. These differences could also be due to the heavily male sample of school 2, which can access condoms without a prescription, while females require a prescription for all commonly used birth control.

Third, findings support exploring the development of a technology based sexual health risk-reduction program through a joint-Facebook, WhatsApp program. This program could (a) reach the greatest number of students on the most frequent basis; (b) include users from the desired age range of 11–18; (c) assure participant anonymity; and (d) quickly distribute health information. Health providers could use the public Facebook portion to upload health information and announce medical student lectures, while providing their WhatsApp messaging information through the Facebook page gives students a secure line for personal inquiries. Though they are used at similar rates, qualitative responses revealed a preference for WhatsApp compared to text messaging. These connections would be established to give students more access to health professionals, and more resources for autonomy in sexual health education.

Differing responses between age groups suggest offering age-specific programs. The write-in survey responses for 15–18 year-olds showed greater specificity of desired information, and these participants were more likely to self-report sexual activity, which could require a different approach. Future studies should evaluate if age-specific programs could be more effective in disseminating sexual health information. Given privacy concerns and higher phone usage among older students, the 15–18-year-old program could develop the private WhatsApp portion, while the 12–14-year-old program would primarily use Facebook.

Per the participants’ recommendation, medical student health lectures could be implemented alongside a technology program. Studies evaluating sexual health education have shown that programs which prioritize empowerment [9,33] and peer mentorship [3,20,26] are more effective than traditional classroom approaches. Introducing positive role models could increase participation, and give medical students opportunities to volunteer and develop skills useful for physicians. School schedules allow for afternoon lectures, which all focus group participants reported interest in attending.

Fourth, the hesitance of focus group participants to respond in front of their peers and conversations with school staff demonstrate a lack of self-confidence and empowerment. Teachers could be more proactive in engaging student discussion, rather than having them passively receive information. By encouraging classroom discussions, students can become more self-confident and self-aware, both of which are crucial for adolescent development and engaging sexual health topics.

It is crucial that the program, though a collaborative between health professionals and students, be owned by the youth. Focus groups revealed that participants lacked areas to socialize outside of school, and research has shown structured after school programs can lead to a reduction in sexual risk-behaviors among adolescents [34]. A program for youth to obtain health information while cultivating social skills could reinforce positive behaviors and discourage negative ones. Empowerment through participation must be key to program design. Active participation avoids the vertical teaching model observed in schools by focusing on student learning outcomes. By requiring participation and focusing on student desires, participants can begin to feel more empowered in their health decisions.

This information combined with the gaps in knowledge suggest that health information should include: pregnancy prevention beyond condoms, obtaining contraceptives, STDs, self-care, autonomy, and empowerment. Designed as a Facebook-WhatsApp system, this program could be highly attractive and frequently utilized by Ecuadorean youth as a reliable source of sexual health knowledge.

There were several limitations to this study. First, the differences among the two sampled populations suggest varied results across schools. Because of this, generalizability of results is limited. Second, this study did not evaluate health providers’ interest, a necessary piece to the proposed program. Future studies should focus on providers’ suggestions, perspectives and desires for such a program. Only a small percentage of students obtained parental consent for participation. This affects sample size, and reflects former studies in Ecuador [35] which suggest the need to engage entire families in sexual health education, as many participants reported barriers to conversations with their parents. Fourth, males were less likely to contribute during focus groups, which could have skewed qualitative data to female perspectives. Future studies could focus on effective methods to engage male adolescents in sexual health discussions.

Despite these limitations, our study adds to the growing literature on technology based sexual health risk-reduction programs [4,25,26,27,28,29,30]. Gaps in health knowledge and reliable sources of health information throughout this study contribute to studies showing a lack of sexual health education among poor communities, thereby increasing risk for adolescent pregnancy and STI transmission [3,16,21]. For the first time, this project studied Latin American adolescents, and engaged participants from the beginning acceptability stages rather than reviewing a program after implementation. Our findings, which show an intense desire from participants to self-design the program, suggest a new approach to the emerging field of technology based education, which engages adolescents throughout all stages of the program. It is crucial to continue to explore this approach, as more technologies are discovered as effective measures to engage adolescents, and as community-based-participatory-research becomes more common [32]. Youth throughout Latin America will continue to be at-risk for unplanned pregnancy, STD transmission and other harmful outcomes, so long as they lack reliable sources of health information. This study provides important implications for the desire, need, and acceptability of a technology-based sexual health risk-reduction program as a legitimate public health intervention for adolescents.

## References

[1] World Health Organization. Adolescent pregnancy. http://www.who.int/mediacentre/factsheets/fs364/en/ Published September 2014. Accessed January 4, 2017.

[2] Guttmacher Institute. Adolescent Pregnancy and Its Outcomes Across Countries. https://www.guttmacher.org/fact-sheet/adolescent-pregnancy-and-its-outcomes-across-countries Published April 6, 2016. Accessed January 4, 2017.

[3] Tebbets C and Redwine D. Beyond the clinic walls: Empowering young people through Youth Peer Provider programmes in Ecuador and Nicaragua. Reproductive Health Matters. 2013; 21(41): 143–153. DOI: 10.1016/S0968-8080(13)41693-2 23684197

[4] Rokicki S, Cohen J, Salomon JA and Fink G. Impact of a Text-Messaging Program on Adolescent Reproductive Health: A Cluster–Randomized Trial in Ghana. American Journal of Public Health. 2017; 107(2): 298–305. DOI: 10.2105/AJPH.2016.303562 27997236 PMC5227930

[5] Ivanova O, Pozo KC, Segura ZE, et al. Lessons learnt from the CERCA Project, a multicomponent intervention to promote adolescent sexual and reproductive health in three Latin America countries: A qualitative post-hoc evaluation. Evaluation and Program Planning. 2016; 58: 98–105. DOI: 10.1016/j.evalprogplan.2016.06.007 PMC4987454 27347640

[6] Hall KS, Richards JL and Harris KM. Social disparities in the relationship between depression and unintended pregnancy during adolescence and young adulthood. Journal of Adolescent Health. 2017; 60(6): 688–697. DOI: 10.1016/j.jadohealth.2016.12.003 28109736 PMC5441928

[7] Yu SH, Mason J, Crum J, Cappa C and Hotchkiss DR. Differential effects of young maternal age on child growth. Global Health Action. 2016; 9(1): 31171 DOI: 10.3402/gha.v9.31171 27852422 PMC5112350

[8] Gómez-restrepo C, Padilla muñoz A and Rincón CJ. A cross-sectional study of school dropout in adolescents: National Mental Health Survey Colombia 2015. Rev Colomb Psiquiatr. 2016; 45(Suppl 1): 105–112. DOI: 10.1016/j.rcp.2016.09.003 27993244

[9] Sandøy IF, Mudenda M, Zulu J, et al. Effectiveness of a girls’ empowerment programme on early childbearing, marriage and school dropout among adolescent girls in rural Zambia: Study protocol for a cluster randomized trial. Trials. 2016; 17(1). DOI: 10.1186/s13063-016-1682-9 PMC5148869 27938375

[10] Whitehead E. Teenage pregnancy: On the road to social death. International Journal of Nursing Studies. 2001; 38(4): 437–446. DOI: 10.1016/S0020-7489(00)00086-9 11470102

[11] SmithBattle LI. Reducing the stigmatization of teen mothers. MCN, The American Journal of Maternal/Child Nursing. 2013; 38(4): 241–243. DOI: 10.1097/NMC.0b013e31829144a2 23571424

[12] Ellison MA. Authoritative knowledge and single women’s unintentional pregnancies, abortions, adoption, and single motherhood: Social stigma and structural violence. Medical Anthropology Quarterly. 2003; 17(3): 322–347. DOI: 10.1525/maq.2003.17.3.322 12974201

[13] Curry M. Effects of abuse on maternal complications and birth weight in adult and adolescent women. Obstetrics & Gynecology. 1994; 92(4): 530–534. DOI: 10.1016/S0029-7844(98)00258-0 9764624

[14] Unicef. Experiences and accounts of pregnancy amongst adolescents Panamá, Republic of Panama: Plan; 2014.

[15] Unicef. Teenage motherhood in Latin America and the Caribbean: Trends, problems and challenges Santiago, Chile; 2007.

[16] Centro de Estudios de Población y Desarrollo Social (CEPAR). Encuesta Demográfica y de Salud Materna e Infantil 2004. World Bank Group; 2004.

[17] Ministerio de Salud. Plan Nacional de Prevención del Embarazo en Adolescentes en Ecuador; 2007.

[18] Mason-Jones AJ, Sinclair D, Mathews C, Kagee A, Hillman A and Lombard C. School-based interventions for preventing HIV, sexually transmitted infections, and pregnancy in adolescents. Cochrane Database Syst Rev. 2016; 11: CD006417 DOI: 10.1002/14651858.CD006417.pub3 27824221 PMC5461872

[19] Gavin L, Catalano R, David-Ferdon C, Gloppen K and Markham C. Positive youth development programs that promote adolescent reproductive health. Journal of Adolescent Health. 2010; 44(2): 75–91. DOI: 10.1016/j.jadohealth.2008.10.038 20172462

[20] Cunningham SD, Kerrigan DL, Jennings JM and Ellen JM. Relationships between perceived STD-related stigma, STD-related shame and STD screening among a household sample of adolescents. Perspectives on Sexual and Reproductive Health. 2009; 41(4): 225–230. DOI: 10.1363/4122509 20444177 PMC4334654

[21] Malek AM. Delay in seeking care for sexually transmitted diseases in young men and women attending a public STD clinic. The Open AIDS Journal. 2013; 7(1): 7–13. DOI: 10.2174/1874613620130614002 24078858 PMC3785038

[22] Adolescent fertility rate (births per 1,000 women ages 15–19). Adolescent fertility rate (births per 1,000 women ages 15–19)|Data. http://data.worldbank.org/indicator/SP.ADO.TFRT?locations=EC Published 2017. Accessed July 5, 2017.

[23] Anderson JE and Lowen CA. Connecting youth with health services: Systematic review. Canadian Family Physician. 2010; 56. 20705886 PMC2920783

[24] GSMA Association. The Mobile Economy: Latin America and the Caribbean 2016 London, United Kingdom; 2016.

[25] Bull S, Devine S, Schmiege SJ, Pickard L, Campbell J and Shlay JC. Text messaging, teen outreach program, and sexual health behavior: A cluster randomized trial. American Journal of Public Health. 2016; 106(S1). DOI: 10.2105/AJPH.2016.303363 PMC5049474 27689478

[26] Devine S, Bull S, Dreisbach S and Shlay J. Enhancing a teen pregnancy prevention program with text messaging: Engaging minority youth to develop TOP Plus text. Journal of Adolescent Health. 2014; 54(3). DOI: 10.1016/j.jadohealth.2013.12.005 24560081

[27] Horvath T, Azman H, Kennedy GE and Rutherford GW. Mobile phone text messaging for promoting adherence to antiretroviral therapy in patients with HIV infection. Cochrane Database of Systematic Reviews; 3 2012 DOI: 10.1002/14651858.CD009756 PMC6486190 22419345

[28] Devine S, Leeds C, Shlay JC, Leytem A, Beum R and Bull S. Methods to assess youth engagement in a text messaging supplement to an effective teen pregnancy program. Journal of Biomedical Informatics. 2015; 56: 379–386. DOI: 10.1016/j.jbi.2015.07.003 26173038

[29] Chavez NR, Shearer L and Rosenthal SL. Use of digital media technology for primary prevention of STIS/HIV in adolescents and young adults: A systematic review of the literature. J Pediatr Adolesc Gynecol; 2013. 10.1016/j.jpag.2013.07.008 24332613

[30] Lim MSC, Hocking JS, Hellard ME and Aitken CK. SMS STI: a review of the uses of mobile phone text messaging in sexual health. Int J STD AIDS. 2008; 19: 287–290. DOI: 10.1258/ijsa.2007.007264 18482956

[31] Graneheim U and Lundman B. Qualitative content analysis in nursing research: Concepts, procedures and measures to achieve trustworthiness. Nurse Education Today. 2004; 24(2): 105–112. DOI: 10.1016/j.nedt.2003.10.001 14769454

[32] O’Toole TP, Aaron KF, Chin MH, Horowitz C and Tyson F. Community-based participatory research. Journal of General Internal Medicine. 2003; 18(7): 592–594. DOI: 10.1046/j.1525-1497.2003.30416.x 12848844 PMC1494882

[33] Cordovez B, Patiño F, Metz S, et al. Linking Comprehensive Maternal Health Care to Empowerment And Better Sexual And Reproductive Health Choices Among Young Mothers: Lessons from Cuenca, Ecuador; 2011.

[34] Thomas A. Examining the effects of afterschool programs on adolescent pregnancy.

[35] Molleda L, Estrada Y, Lee TK, et al. Short-term effects on family communication and adolescent conduct problems: Familias Unidas in Ecuador. Prevention Science; 12 2016 DOI: 10.1007/s11121-016-0744-2 27981448

